# Supplementary material for: Precise Identification of Chromosome Constitution and Rearrangements in Wheat–Thinopyrum intermedium Derivatives by ND-FISH and Oligo-FISH Painting
Source: Plants (Basel). 2022 Aug 13;11(16):2109. doi: 10.3390/plants11162109 (PMC9415406; doi:10.3390/plants11162109)
Supplement: Supplementary file 1 [file plants-11-02109-s001.zip › plants-1861651-supplementary.pdf]

Table S1. The genome-specific oligo probes for distinguishing the Triticeae species by ND-FISH

Name	Sequences	Species	Genome(s)	References
Oligo-k288	CTTCATAGTCCGGGAGTCCGGCC AAAGGTCATAGTCCGGCCATCC	*T. turgidum*	A, B	Wang et al. 2019
Oligo-D	TACGGGTGCCAAACGAGTGTCTG AAAGACTCCTCGAGAGGAAAAT GCGAA	*Aegilops tauschii*	D	Tang et al. 2018
Oligo-Ku	GATCGAGACTTCTAGCAATAGGC AAAAATAGTAATGGTATCCGGG TTCG	*Secale*, *Dasypyrum*	R, V	Xiao et al. 2017
Oligo-pDb12H	TCAGAATTTTTAGGATAGCAGAA GTATTCGAAATACCCAGATTGCT ACAG	*Dasypyrum*	V, V^b, J^S	Yu et al. 2019, 2021
Oligo-B11	TCCGCTCACCTTGATGACAACAT CAGGTGGAATTCCGTTCGAGGG	*Thinopyrum, Dasypyrum, Pseudoroegneria*	St, E, J J^S, V^b, Ns	Xi et al. 2019
Oligo-5SrDNA	TCAGAACTCCGAAGTTAAGCGTG CTTGGGCGAGAGTAGTAC	All Triticeae species	A, B, D, St, E, J J^S, V^b, Ns	Yu et al. 2019
Oligo-pTa71	GGGCAAAACCACGTACGTGGCA CACGCCGCGTA	All Triticeae species	A, B, D, St, E, J J^S, V^b, Ns	Tang et al. 2014
Oligo-3A1	AATAATTTTACACTAGAGTTGAA CTAGCTCTATAAGCTAGTTCA	All Triticeae species	A, B, D, St, E, J J^S, V^b, Ns	Lang et al. 2019
Oligo-pSt122	GGCTCACATTAGGGAAGAATCG GTGAACAAAGAAAAGACAAATT CACCGTATAGAG	*Thinopyrum, Dasypyrum, Pseudoroegneria*	St, E, J J^S, V^b, Ns	Li et al. 2015

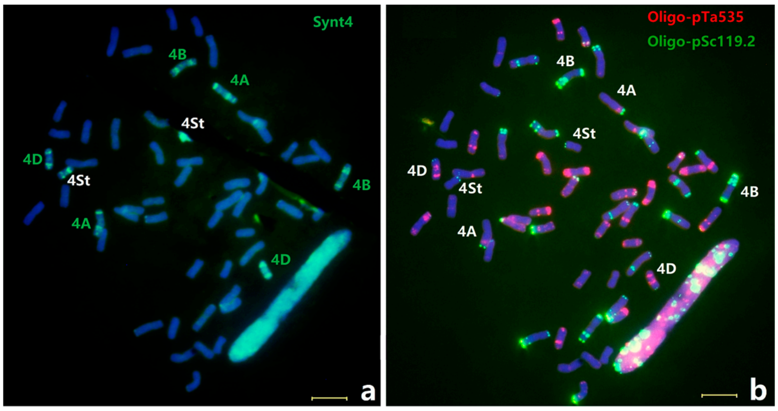

Figure S1. Sequential Oligo-FISH painting using specific bulked oligo probes Synt4 **(a)** and FISH probes Oligo-pSc119.2 +Oligo-pTa535 (**b**) for line 78829.

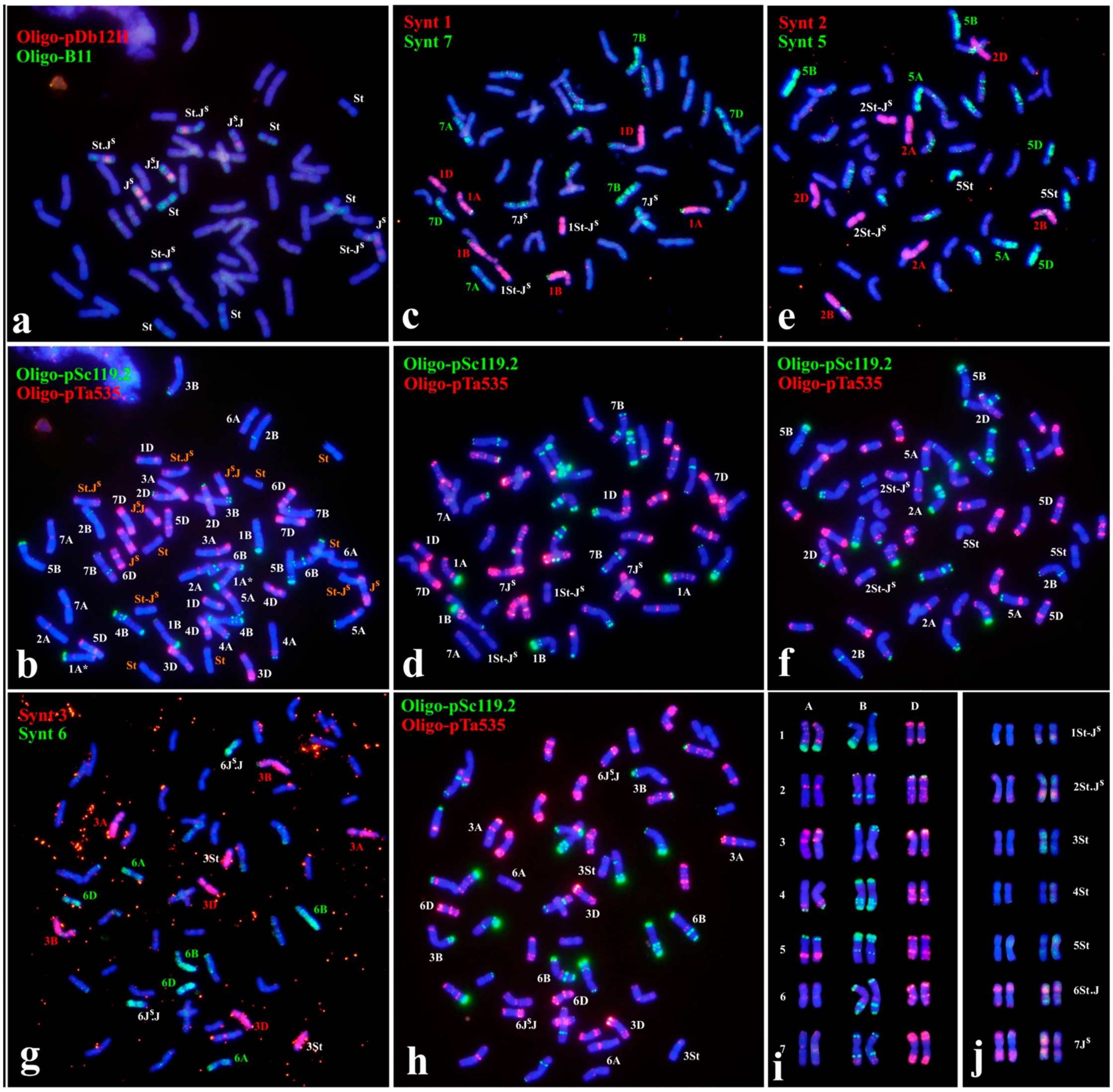

Figure S2 Karyotyping of 12 wheat-*Thinopyrum intermedium* partial amphiploids by sequential ND-FISH and Oligo-FISH painting.

Figure S2-1. Karyotyping of the partial amphiploid 78784 by sequential ND-FISH (a, b, d, f, h) and Oligo-FISH painting (c, e, g). Karyotypes of wheat chromosomes (i) and Th. intermedium (j) were showed, respectively.

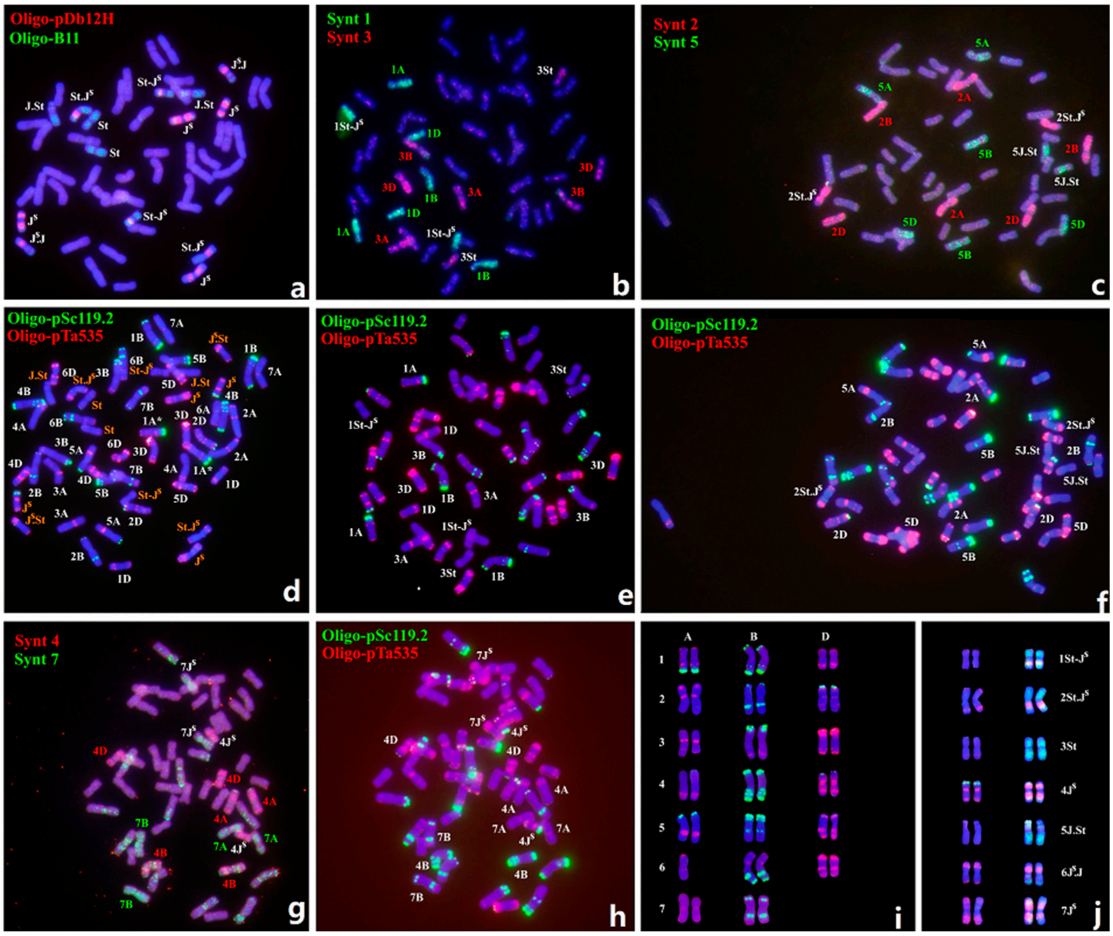

Figure S2-2. Karyotyping of the partial amphiploid 8024 by sequential ND-FISH (a, b, d, f, h) and Oligo-FISH painting (c, e, g). Karyotypes of wheat chromosomes (i) and Th. intermedium (j) were showed, respectively

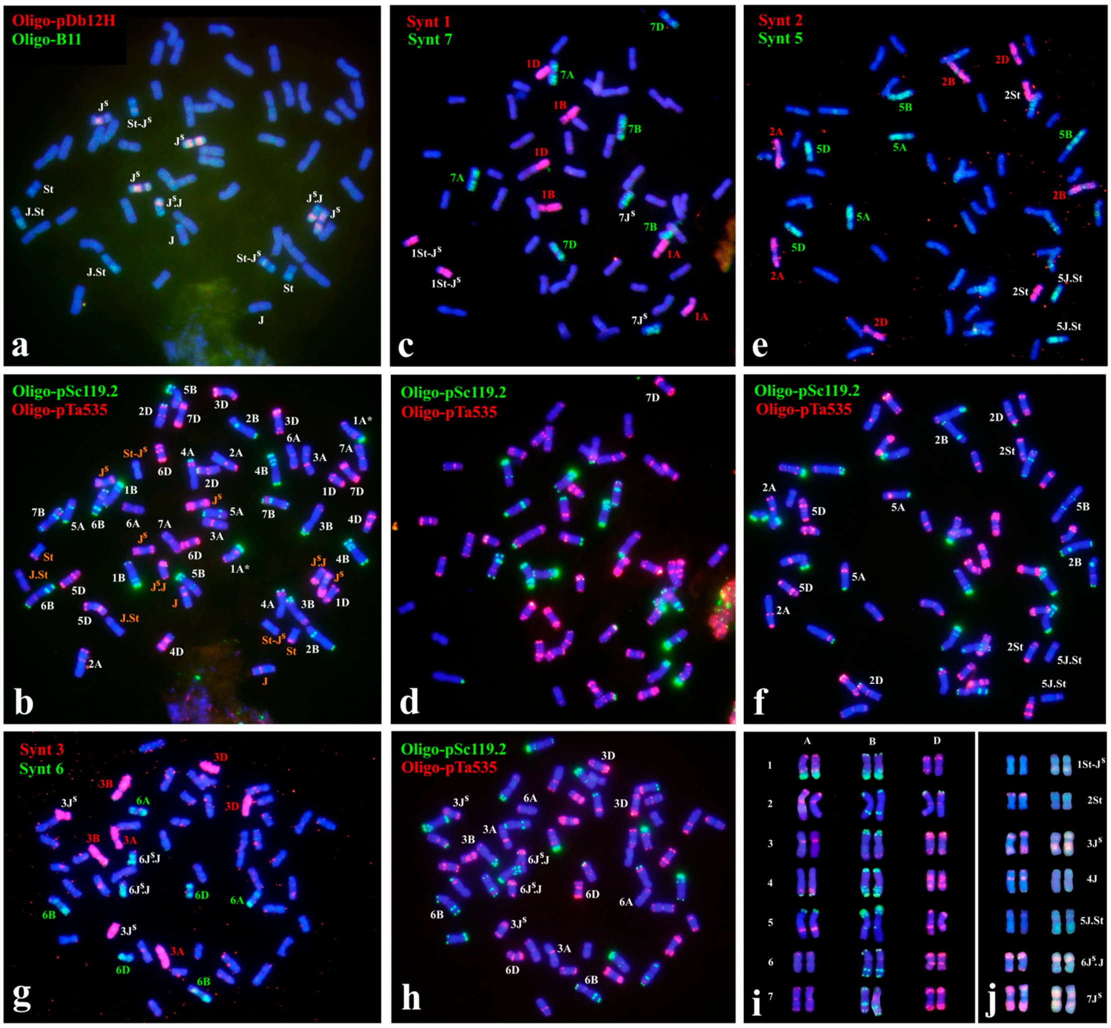

Figure S2-3. Karyotyping of the partial amphiploid TAI7045 by sequential ND-FISH (a, b, d, f, h) and Oligo-FISH painting (c, e, g). Karyotypes of wheat chromosomes (i) and Th. intermedium (j) were showed, respectively.

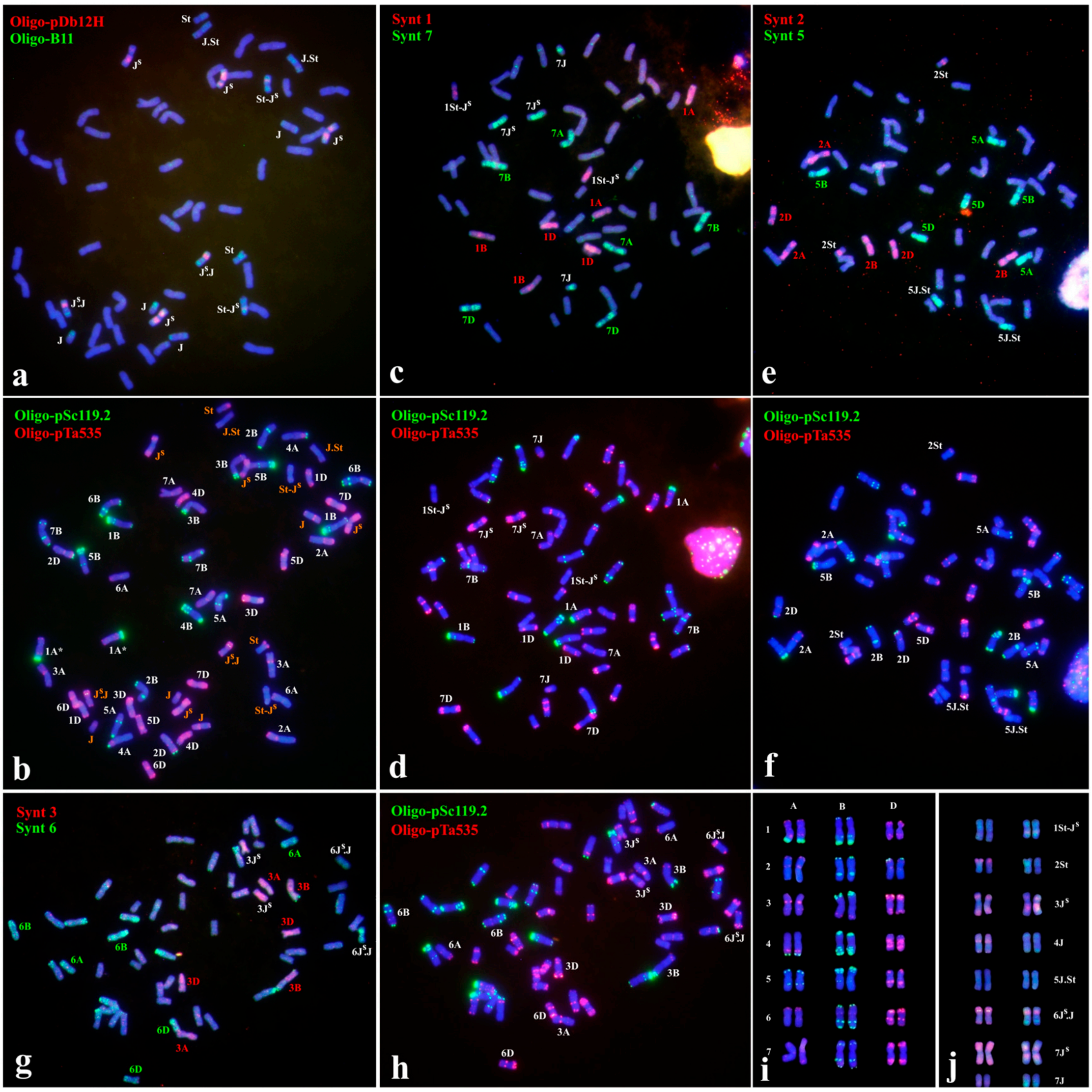

Figure S2-4. Karyotyping of the partial amphiploid TAI7047 by sequential ND-FISH (a, b, d, f, h) and Oligo-FISH painting (c, e, g). Karyotypes of wheat chromosomes (i) and Th. intermedium (j) were showed, respectively.

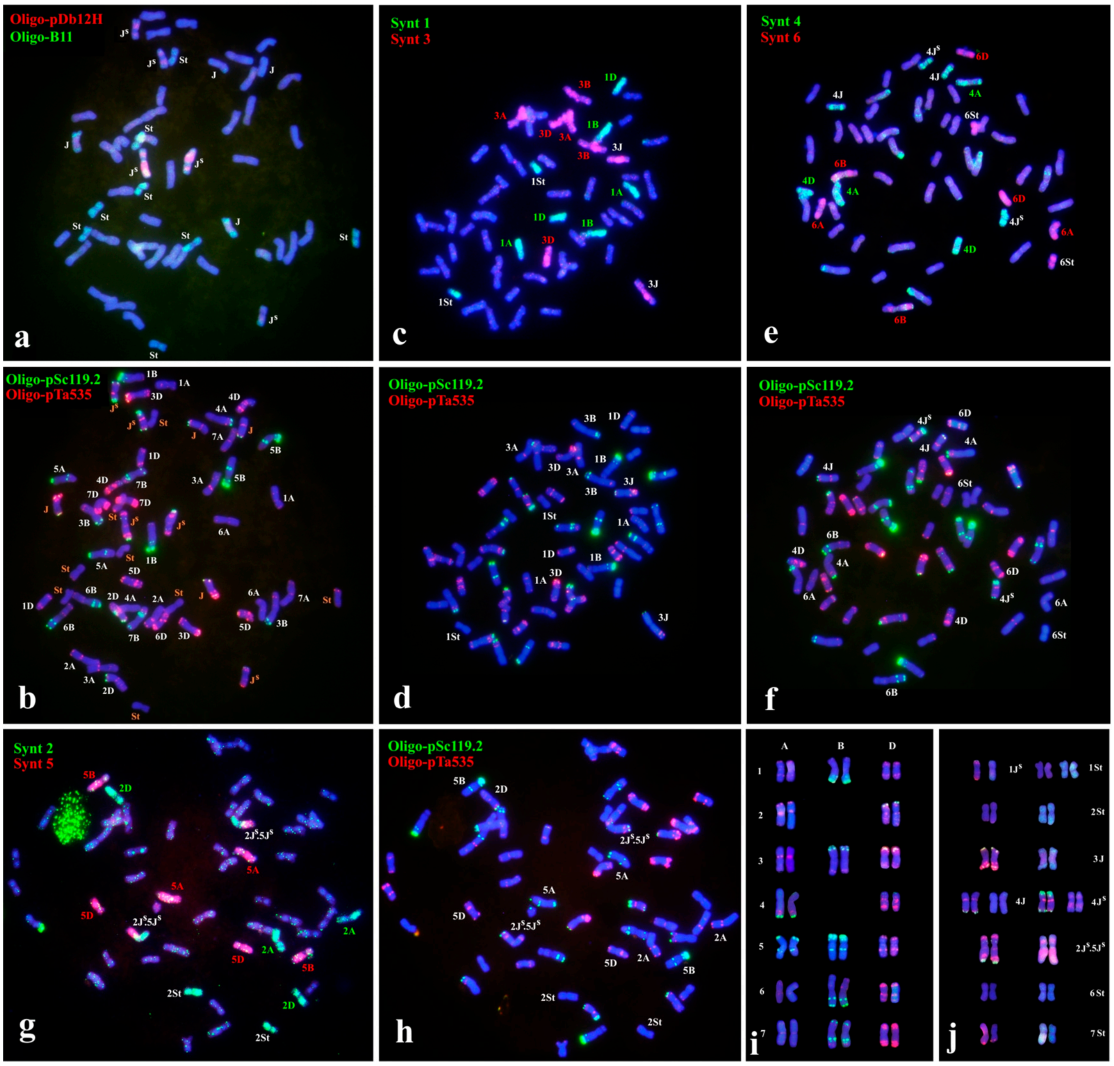

Figure S2-5. Karyotyping of the partial amphiploid TE-1502 by sequential ND-FISH (a, b, d, f, h) and Oligo-FISH painting (c, e, g). Karyotypes of wheat chromosomes (i) and Th. intermedium (j) were showed, respectively.

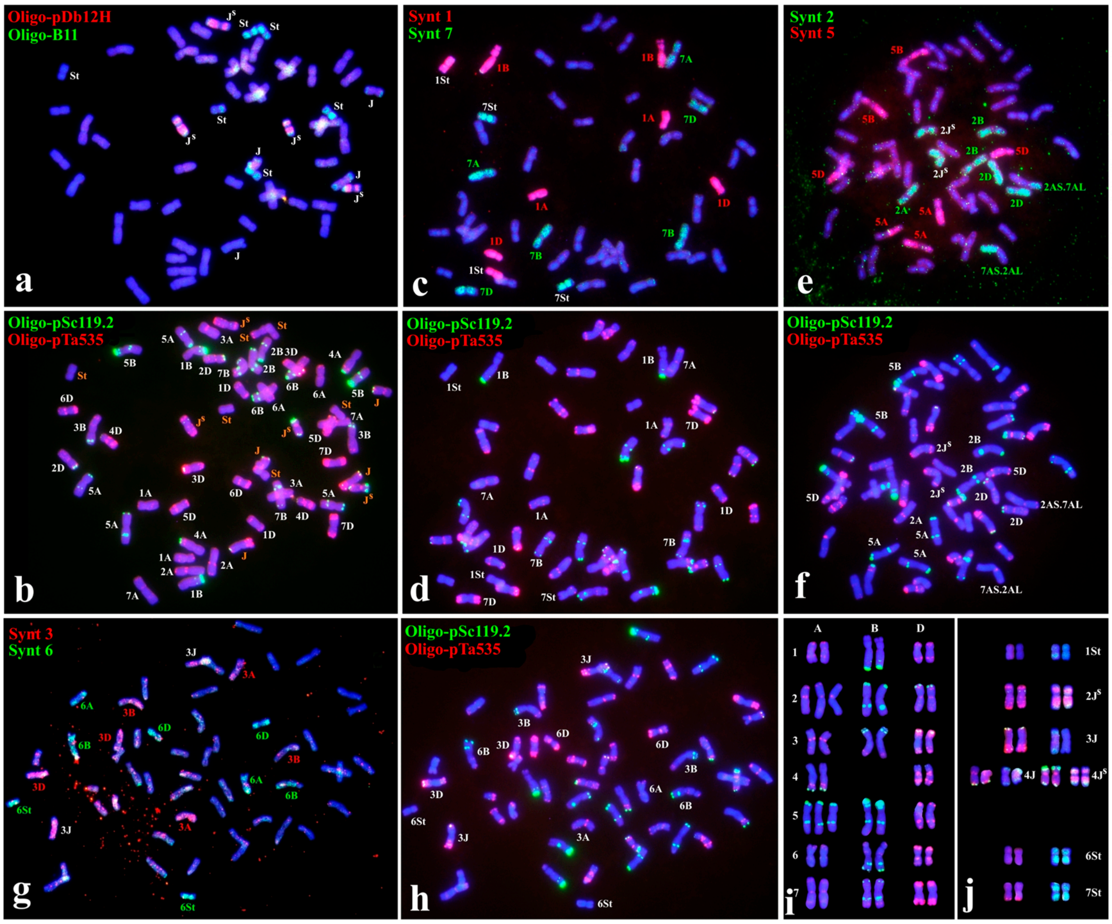

Figure S2-6. Karyotyping of the partial amphiploid TE-1508 by sequential ND-FISH (a, b, d, f, h) and Oligo-FISH painting (c, e, g). Karyotypes of wheat chromosomes (i) and Th. intermedium (j) were showed, respectively.

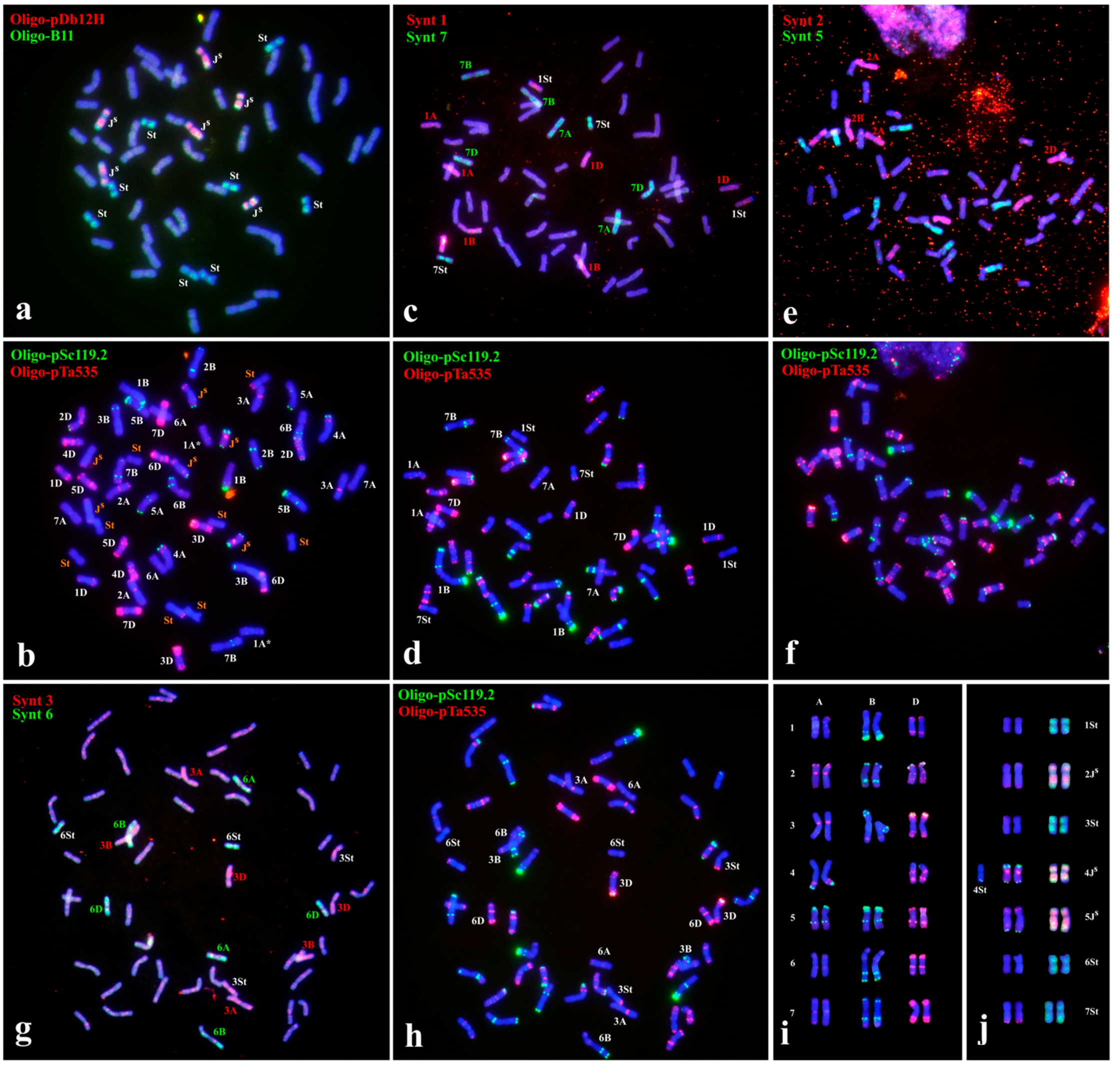

Figure S2-7. Karyotyping of the partial amphiploid TH101-2 by sequential ND-FISH (a, b, d, f, h) and Oligo-FISH painting (c, e, g). Karyotypes of wheat chromosomes (i) and Th. intermedium (j) were showed, respectively.

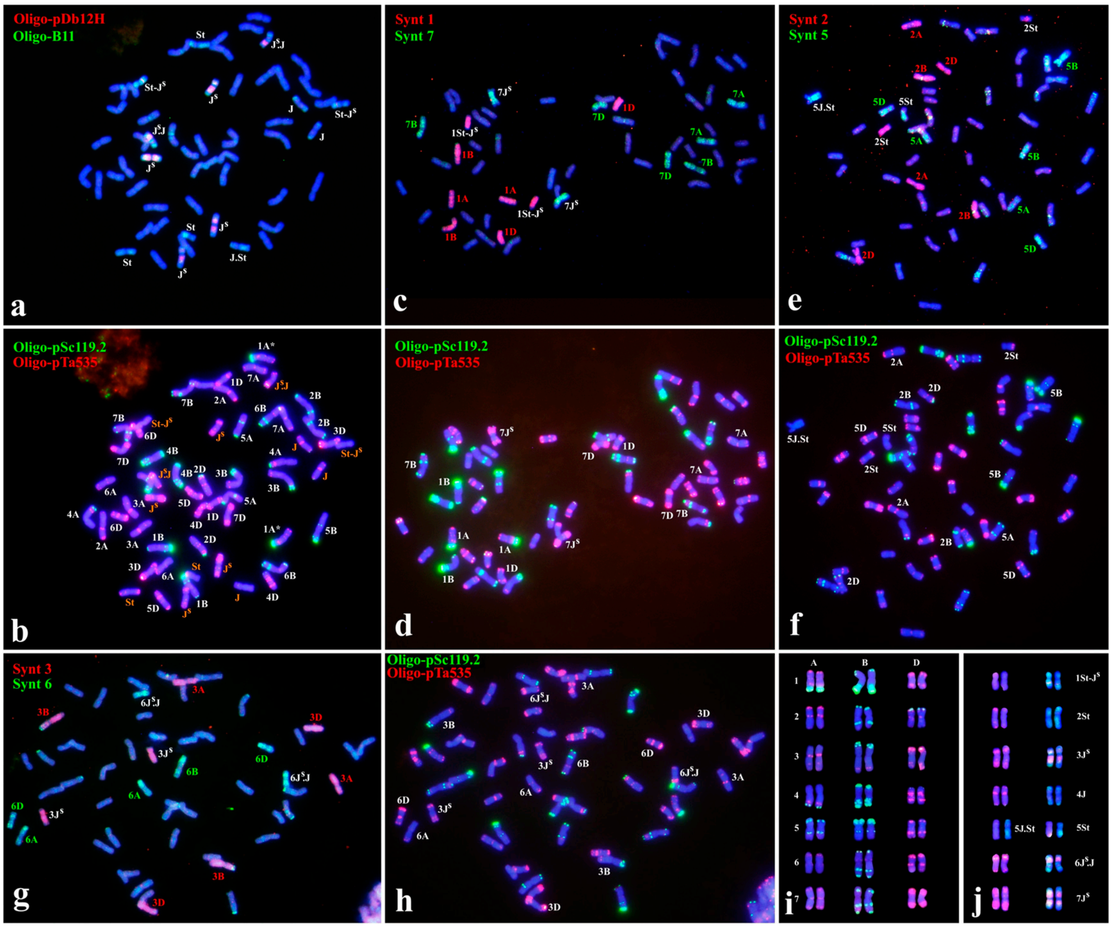

Figure S2-8. Karyotyping of the partial amphiploid y70-1-4 by sequential ND-FISH (a, b, d, f, h) and Oligo-FISH painting (c, e, g). Karyotypes of wheat chromosomes (i) and Th. intermedium (j) were showed, respectively.

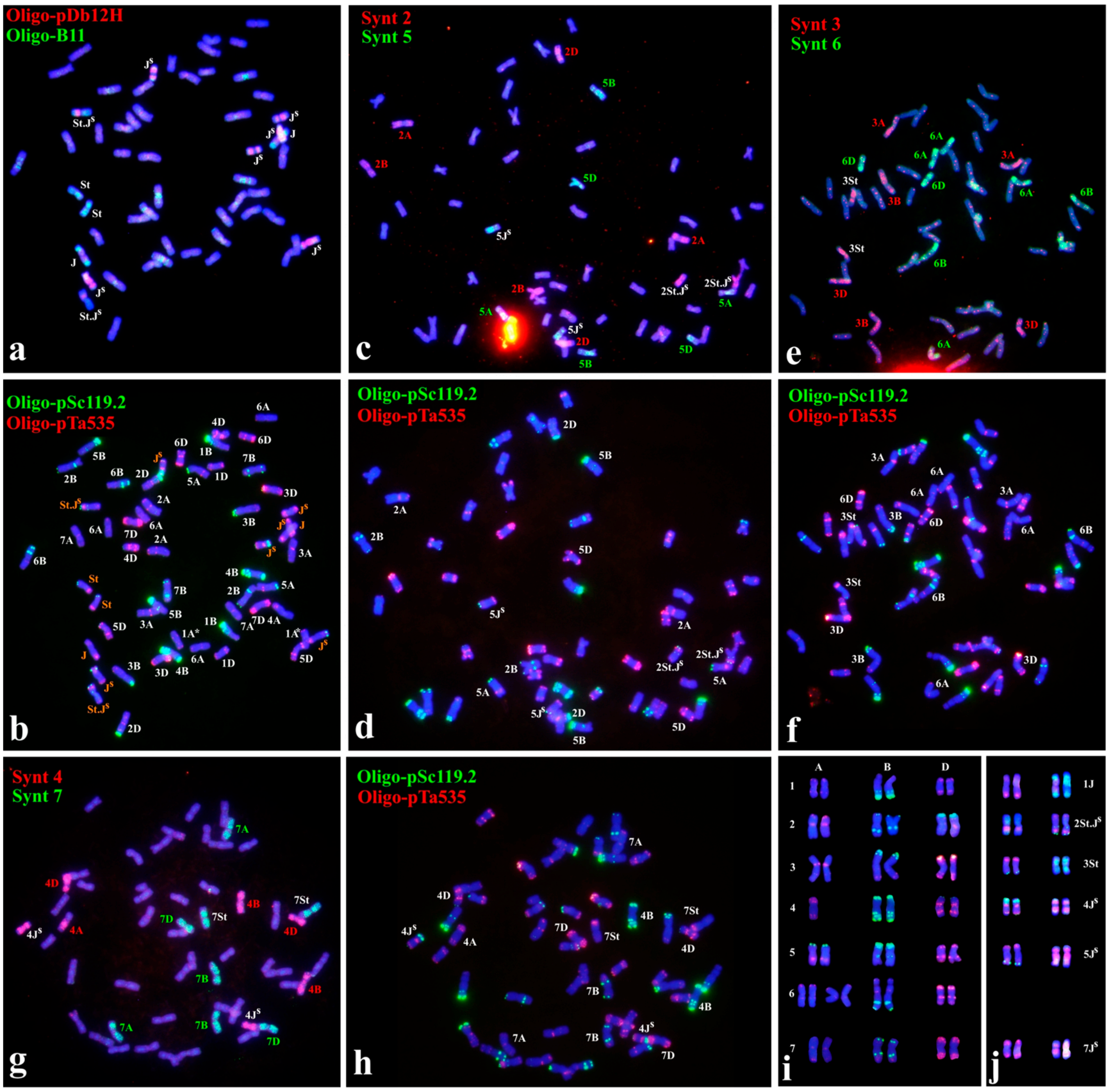

Figure S2-9. Karyotyping of the partial amphiploid Zhong2 by sequential ND-FISH (a, b, d, f, h) and Oligo-FISH painting (c, e, g). Karyotypes of wheat chromosomes (i) and Th. intermedium (j) were showed, respectively.

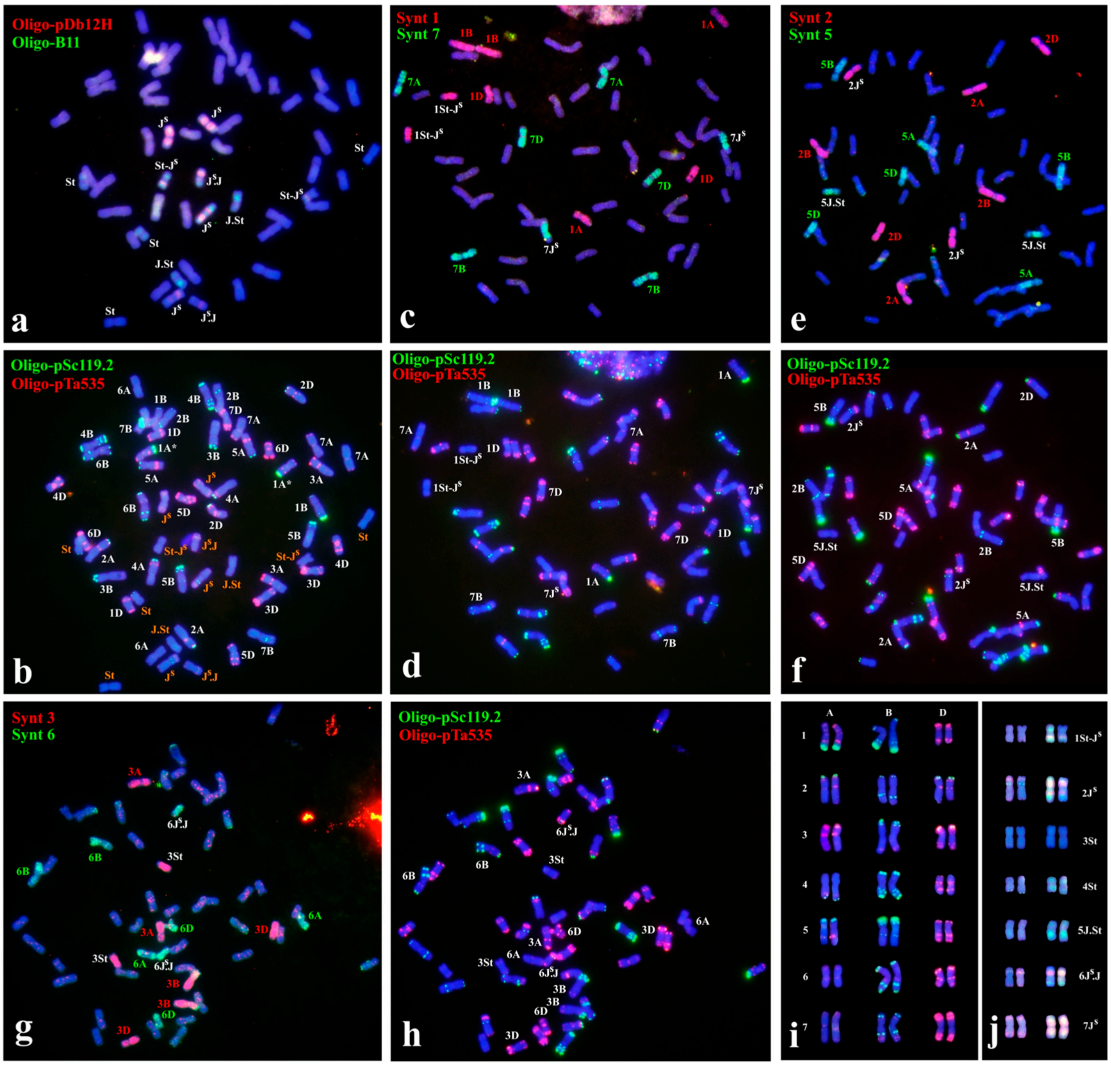

Figure S2-10. Karyotyping of the partial amphiploid Zhong 3 by sequential ND-FISH (a, b, d, f, h) and Oligo-FISH painting (c, e, g). Karyotypes of wheat chromosomes (i) and Th. intermedium (j) were showed, respectively.

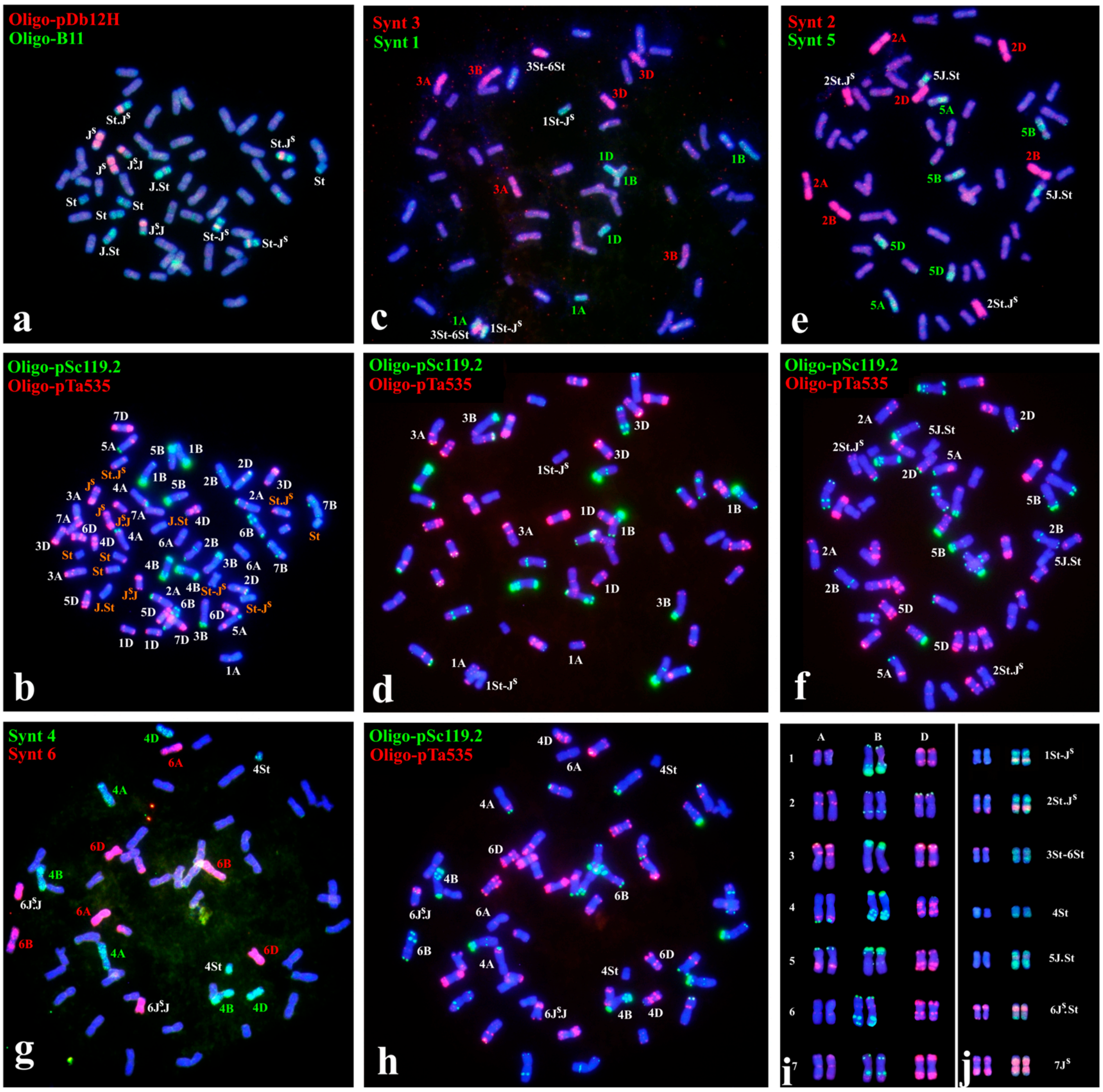

Figure S2-11. Karyotyping of the partial amphiploid Zhong 4 by sequential ND-FISH (a, b, d, f, h) and Oligo-FISH painting (c, e, g). Karyotypes of wheat chromosomes (i) and Th. intermedium (j) were showed, respectively.

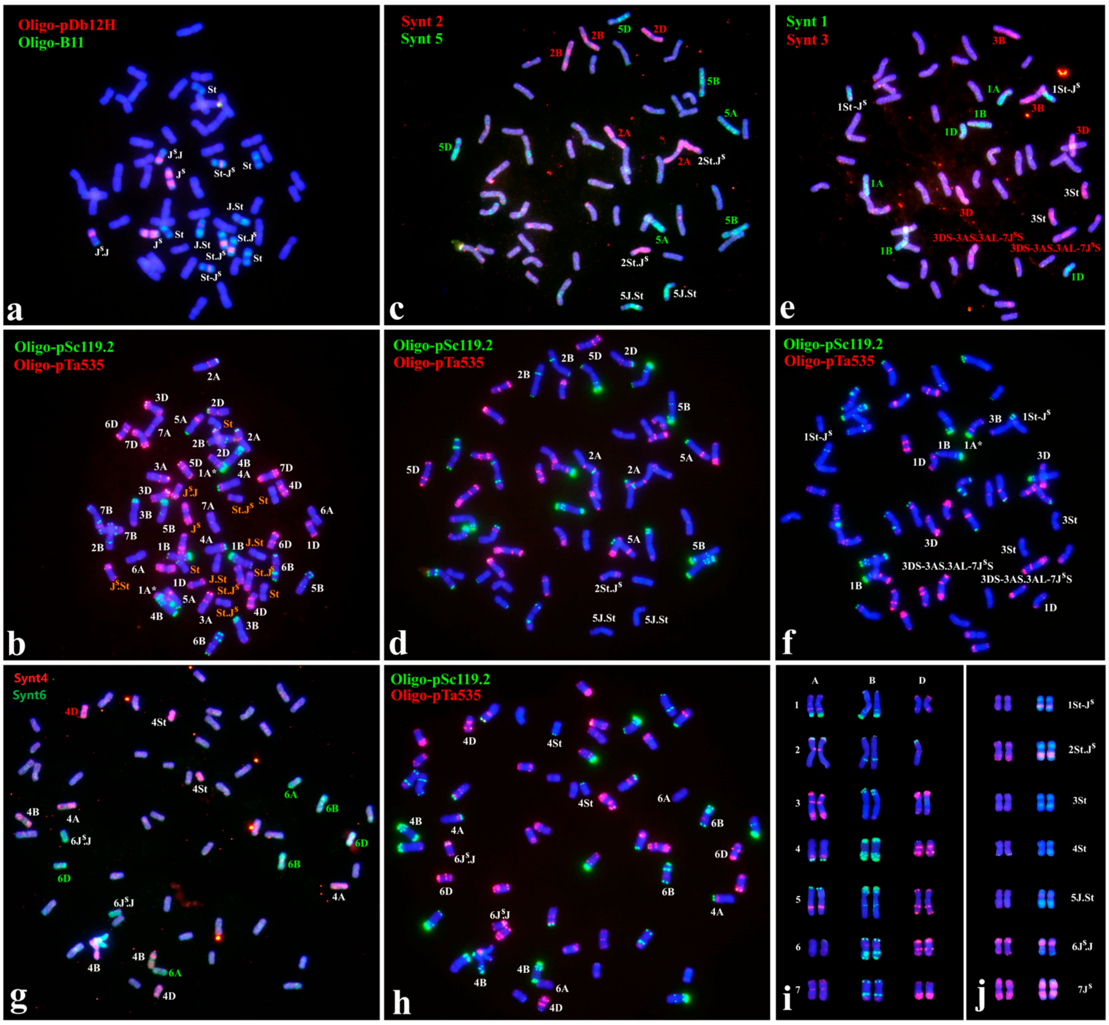

Figure S2-12. Karyotyping of the partial amphiploid Zhong 5 by sequential ND-FISH (a, b, d, f, h) and Oligo-FISH painting (c, e, g). Karyotypes of wheat chromosomes (i) and Th. intermedium (j) were showed, respectively.

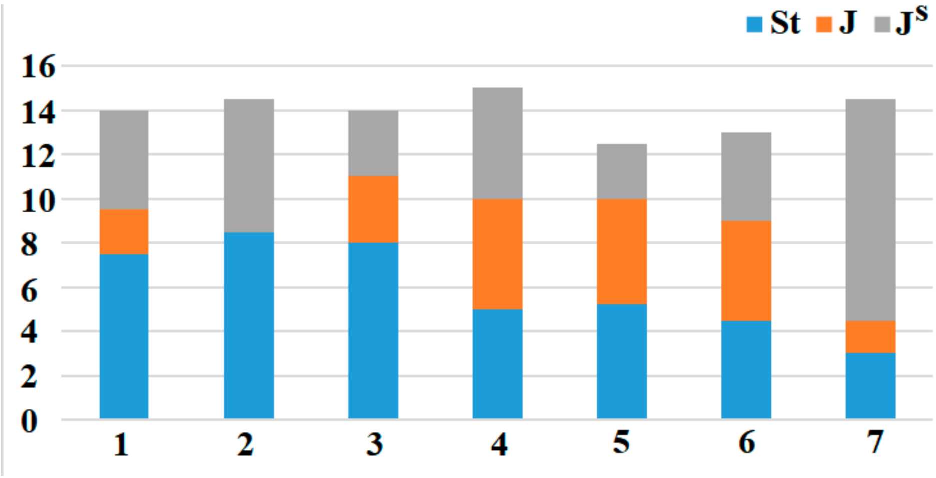

Figure S3. The frequency of St, J and J^S chromosomes in the identified wheat-*Th. intermedium* partial amphiploid.

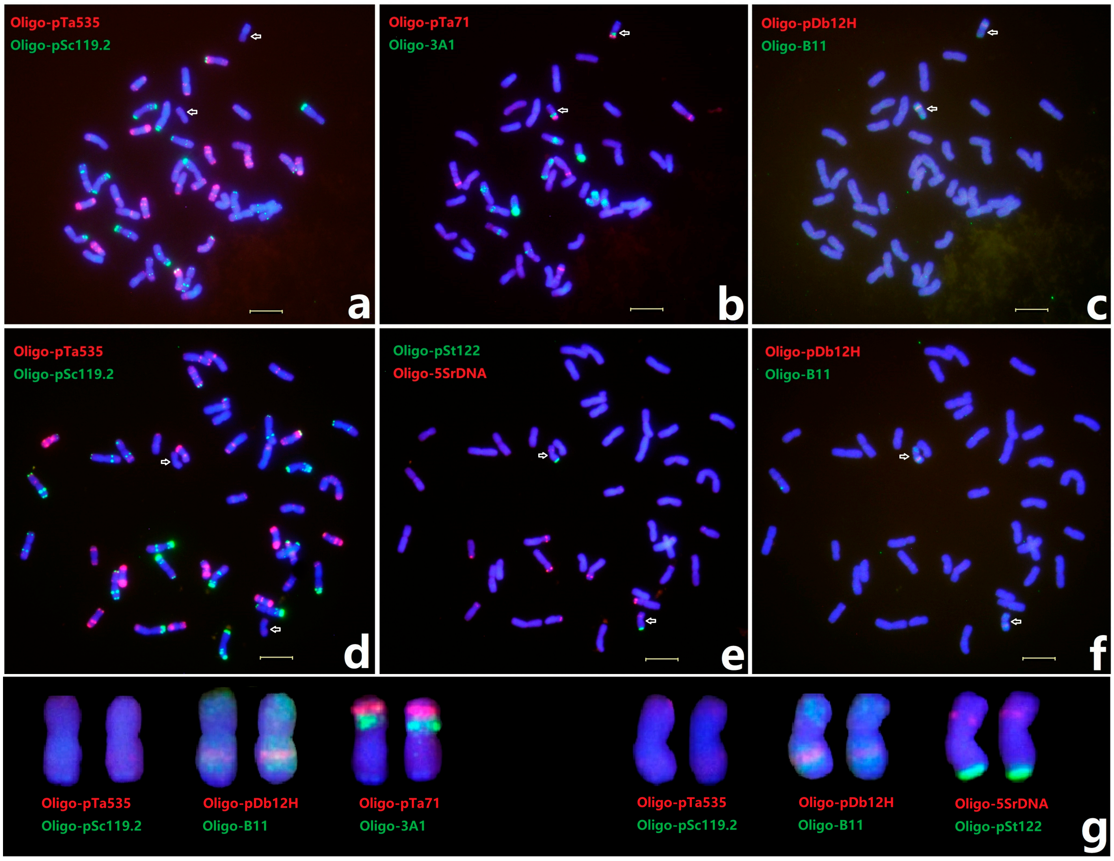

Figure S4. Sequential ND-FISH of Z3 using multiple probes. The probes Oligo-pSc119.2 (green) + Oligo-pTa535 (red) (**a, d**), Oligo-pTa71 (red) + Oligo-3A1 (green) (**b**), Oligo-pDb12H (red) + Oligo-B11(green) (**c, f**), Oligo-pSt122(green) + Oligo-5SrDNA (red) (**e**) are indicated. The additional *Th. intermedium* chromosomes in Z3 are shown (**g**). Bars, 10 μm.
